# Exploration of a Capability-Focused Aerospace System of Systems Architecture Alternative with Bilayer Design Space, Based on RST-SOM Algorithmic Methods

**DOI:** 10.1155/2014/536462

**Published:** 2014-03-24

**Authors:** Zhifei Li, Dongliang Qin, Feng Yang

**Affiliations:** College of Information System and Management, National University of Defense Technology, Changsha, Hunan 410073, China

## Abstract

In defense related programs, the use of capability-based analysis, design, and acquisition has been significant. In order to confront one of the most challenging features of a huge design space in capability based analysis (CBA), a literature review of *design space exploration* was first examined. Then, in the process of an aerospace system of systems design space exploration, a bilayer mapping method was put forward, based on the existing experimental and operating data. Finally, the feasibility of the foregoing approach was demonstrated with an illustrative example. With the data mining RST (rough sets theory) and SOM (self-organized mapping) techniques, the alternative to the aerospace system of systems architecture was mapping from P-space (performance space) to C-space (configuration space), and then from C-space to D-space (design space), respectively. Ultimately, the performance space was mapped to the design space, which completed the exploration and preliminary reduction of the entire design space. This method provides a computational analysis and implementation scheme for large-scale simulation.

## 1. Introduction

Recently, capability-based analysis, design, and acquisition have had a significant impact in defense related programs. The paradigm shift to capabilities-based acquisition is causing a fundamental shift in the way defense-related systems are both engineered and purchased. New mission needs and technological advancements have led to novel directives that are causing defense acquisition planning to utilize a capability-based approach. In particular, advancements in communication and transportation, combined with new and diverse enemies, have led to a call for increased joint operations, more integrated operations, and a better method of designing and acquiring systems and SoS (system of systems) to support these needs.

This capability-based mentality shares a natural link with architecting, in that capabilities are achieved through a series of activities. These activities can be represented as an operational architecture. Through the architecting process, they can be mapped to candidate solutions, which can then be evaluated and compared. These solutions provide the* ways and means* by which a capability is achieved. This kind of approach has been suggested to help address high level capability needs and help avoid the stove piping that has often plagued defense acquisition [1].

The challenge presented by the sheer number of possible alternatives is compounded in SoS problems. In fact, not only is the number of alternatives extremely large, but the alternatives also vary in their specifications, including alternatives across all aspects of the DOTMLPF (doctrine, organization, training, materiel, leadership, people, and facilities) spectrum. It is difficult to gather enough information early on to make an informed decision, but it is also difficult to even determine the criteria by which two extremely different solutions can be compared. Even justifying the acquisition of a new system can be difficult, because it must be shown that the same mission level cannot be achieved with a new arrangement or new uses of existing systems. To further illustrate this challenge, consider a simple mission, which is comprised of completing 10 activities. Then consider that these activities can be performed in two different sequences, thus creating two operational alternatives. Furthermore, each activity can be performed by one of three candidate systems. Three possible organizations could be responsible for conducting this mission and, last, consider that there are two types of networks being considered for enabling communication in the architecture. There are then 2 organizational alternatives ×3^10^ system alternatives ×3 organizational alternatives ×2 network alternatives, resulting in a total of 708,588 alternatives.

Thus, there are several criteria for a design space exploration method for CBA. First, it must be able to capture and define the large number of architectural alternatives available for consideration during the early phases of acquisition and systems engineering. Next, it must provide a way to filter through the design space and find only the promising alternatives for evaluation, while eliminating those that are either unrealistic or are not expected to meet mission goals. Finally, because even the filtering processes will still leave large numbers of alternatives to be evaluated, there must be a way to quickly and accurately evaluate the remaining alternatives.

## 2. Literature Review

Currently, the research of aerospace system of systems architecture alternatives for design space exploration focuses mainly on the* design of the experiment*, the* approximation model*, and* optimization algorithms*.

### 2.1. Design of Experiment

*Design of the experiment* [2] is a mathematical method of statistical analysis that allows for the study of the development of a reasonable alternative using data space technology. DOE has become an indispensable tool in computer-aided design optimization [3]. The main DOE methods include Monte Carlo sampling (MCS) [4], Latin hypercube sampling (LHS) [5], orthogonal array sampling (OA) [6], D-optimal design (DO) [7], and uniform design (UD) [8].

### 2.2. Approximation Model

In order for large-scale computing to simplify the design space and to generate a full understanding of space exploration, especially for large-scale multidisciplinary design space exploration and optimization, the* approximation model* was introduced into the design process. The main approximation models are the* response surface model* (RSM) [9], the* radial basis function neural network* (RBFNN) [10], and the* kriging model* [11].

### 2.3. Optimization Algorithm

In engineering design, optimization algorithms are often used to search among global optimal solutions in the design space; the method can be divided into two categories:* exact methods* and* approximation methods*. The exact methods include* branch and bound* [12],* mathematical programming* [13], and* coordination decomposition* [14]. The exact methods can be proven to be the optimal global solution but are only capable of solving smaller problems. The approximation methods can obtain a solution quickly in large-scale problems but cannot ensure that the resulting solution is optimal [15].

### 2.4. Comparative Analysis

DOE is an essential basic experimental approach in engineering design optimization, which represents the performance of the design space through different distributions of sampling points. However, while the DOE method is capable of sampling within the developed design space and then analyzing on the sampled points, it cannot explore the design space through the sampling itself nor can it divide or reduce the scope of the design space.

As mentioned earlier, design space exploration is one of the application directions of the* approximation model*. Approximate models, however, require repeated sampling when used in design space exploration problems, which will increase the load of computation. At the same time, there are no design space exploration methods that are suitable for the aerospace system design process.

Optimization algorithms of design space exploration, which belong to the latest developments in design optimization, can be used to explore and optimize the design space to find the global optimal solution or a feasible solution. The costs and computational load of the* optimization algorithm* for large-scale design space exploration are very high and inappropriate for an aerospace system of systems design and optimization in the early phase.

Above all, we can see that there is a lack of effective methods to utilize various existing experimental and historical data, as well as data from aerospace SoS, leaving a need for knowledge-based design space exploration methods as a guide for system design optimization. For one thing, since a large amount of computer technology and simulation software in engineering applications is required for the process of aerospace SoS design, when there are large numbers of simulations and experiments, there will be massive amounts of data stored in the data warehouse. It is important to take advantage of this useful data for subsequent SoS design optimization and to then support aerospace SoS design space exploration. Secondly, the existing design space exploration methods are used to approximate and explore directly within the aerospace system design space. In the early phase, however, there is typically a lot of uncertainty and a definite lack of knowledge. The existing methods have a too large computational load and cannot hold up to the design practices and processes. It is imperative to guide the designer to focus on the design space area of concern.

## 3. Proposed Approach

### 3.1. The General Framework of the Method

**Figure 1:**
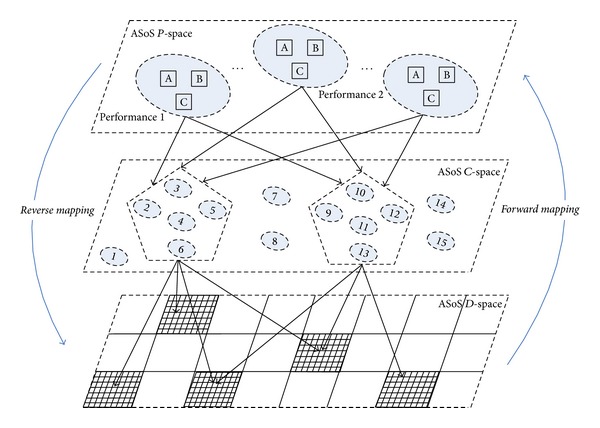
The general framework of the method.

Traditional aerospace SoS optimization is a process that flows from the design space to the performance space, called “*Forward Mapping.*” However, successful experiences and experimental data are difficult to use in the design and development process. Additionally, acquisition staffs tend to pay more attention to the overall SoS performance, hoping to map the route from the performance space (the actual SoS performance and performance evaluation results) to the design space, in order to complete the design space exploration, which can help accurately locate the design space area of concern. Limiting the aerospace SoS design optimization to a smaller space saves time spent searching in an unnecessary area, making the whole design optimization more targeted. Mapping from the “performance space” to the“design space,”referred to here as “*reverse mapping,*” complies with the general rules of aerospace equipment acquisition, as shown in Figure 1.

### 3.2. Bilayer Exploration Process

**Figure 2:**
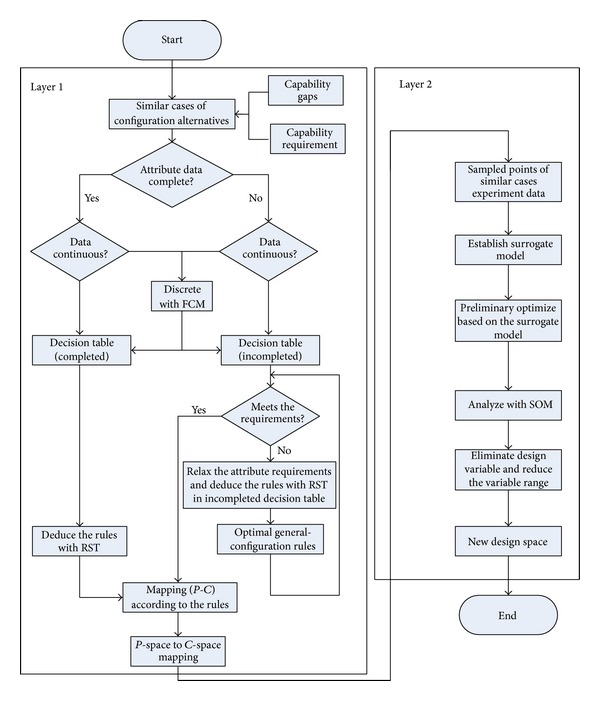
The Bilayer exploration process.

*Layer 1: RST-Based Mapping from P-Space to C-Space.* As shown in Figure 2, this paper studied the aerospace system of systems design space exploration methods of the architecture alternatives, primarily learning from previous design experience to better guide the overall design optimization with use of RST reasoning, based on the analysis of similar cases.

Similar, relevant cases are first selected, according to the capability gap and required operational activities, in order to determine the initial aerospace system configuration, which provides foundational data for subsequent derivation of configuration rules. Secondly, it must be determined whether or not the parameter attributes are complete. Thirdly, if the attribute data of the configuration program is complete, then the configuration rules from the complete configuration decision table are derived, using RST. If incomplete data is included, then reasoning with corresponding use of RST in the incomplete configuration decision table is utilized.

In the process of complete rule reasoning, the selected attributes are first analyzed and the continuous data is discretized, using the FCM (fuzzy C-means) algorithm, which preprocesses data for the use of RST. Secondly, in accordance with the selected configuration, similar cases are collected from the corresponding performance estimates, along with a variety of configuration attribute data, constituting a configuration decision table. Again, the simplest related configuration rules from the configuration decision table are acquired with RST. Finally, when the performance space and the configuration space are positioned corresponding to configuration rules, the mapping from P-space to C-space can be completed.

In the incomplete configuration reasoning process, discretized continuous data must first be put into an incomplete configuration scheme. In accordance with the selected configuration, similar cases can be collected in the corresponding performance estimates, along with a variety of configuration attribute data, marking any uncertainties or missing data in the configuration alternatives with an “∗.” The configuration decision table can then be compiled. Again, due to the incomplete data, there will be uncertain causality. The optimal configuration rules can thus be determined with the similarity function in Section 3.3. The optimized configuration rules should be assessed. If the rules meet the design specifications and system requirements of the design staff, the performance space and the configuration space can be positioned according to the configuration rules, completing the mapping from P-space to C-space. If not, the requirements of attribute decision can be relaxed, and the optimized generalized configuration rules can then be calculated with the optimization of general configuration rule functions, as defined in Section 3.3. Once again, the new optimal general configuration rules must be assessed to determine whether they meet the design specifications and requirements. If so, the iteration is terminated. Finally, according to the configuration rules, positioning the performance space, and the configuration space area according to configuration rules, the mapping from P-space to C-space can be completed.

*Layer 2: SOM-Based Mapping from C-Space to D-Space.* Upon completion of the preliminary configuration of aerospace SoS, relevant experimental data or the actual running information can primarily be selected from similar cases, according to the given aerospace systems within the configuration. Secondly, the relevant* surrogate models* can be established, using relevant information and data, and then preliminary optimization can be made based on the model. Again, the design variables and the objective functions were analyzed using the SOM. A detailed study of the relationship between design variables and the objective function can then be made, using the color changes of a two-dimensional hexagonal grid, eliminating the unimportant design variables and reducing the associated interval of design variables. Finally, the dimensions and the design variables of concern can be determined for the design space and then a new design space can be constructed with a smaller design optimization range than the original, including local and global optimums. The smaller range of a more targeted and relatively transparent design space optimization can improve efficiency, saving design time, and cost.

### 3.3. RST-Based Exploration Algorithm

#### 3.3.1. Aerospace System C-Space Modeling

Aerospace system configuration can be defined as

(1) $$\begin{array}{c} S\;\left(U,A,V,f\right), \end{array}$$

where *U* is a nonempty set of alternatives, *A* is a set of nonempty attributes of a selected configuration, *V* is the range of *α*, *α* ∈ *A*, and *f* is an information function, *f*: *U* → *V*_*α*_, giving each attribute of each object an information value, where *α* ∈ *A*, *x* ∈ *U*, and *f*(*x*, *a*) ∈ *V*_*α*_.

The decision table for aerospace SoS C-space and P-space is defined as follows:

(2) $$\begin{array}{c} S=\left(U,A\cup \left\{d\right\},V,f\right), \end{array}$$

where *U*, *A*, *V*, and *f* have the same meaning within the configuration space model and {*d*} is a decision attribute. The entire aerospace SoS performance space is divided through the actual aerospace system and the user evaluation. Therefore, designers can get {*d*} attribute values from the performance space. 

#### 3.3.2. The Definition of Upper and Lower Approximation in the C-Space

In the aerospace SoS configuration model, each attribute subset *M*⊆*A*, IND(*M*) expresses metarelations between any two configuration alternatives, called indiscernible relations, which are defined as follows:

(3) $$\begin{array}{c} \text{IND}\left(M\right)=\left\{\left(x,y\right)\in U\times U\;|\;\forall \alpha \in M,\alpha \left(x\right)=\alpha \left(y\right)\right\}, \end{array}$$

where *M*⊆*A* (*M* is a subset of the entire attribute *A*) and *X* is a subset of all optional configurations, *U*.

For *X*, the upper and lower approximation of *M* is defined as

(4) $$\begin{array}{c} \underline{M}X=\cup \left\{Y\in \frac{U}{\text{IND}\left(M\right)}\;|\;Y\subseteq X\right\},\\ \bar{M}X=\cup \left\{Y\in \frac{U}{\text{IND}\left(M\right)}\;|\;Y\cap X\neq \emptyset \right\}. \end{array}$$

As seen from the definitions, for the selected configuration *X*, the lower approximation represents the minimum optional configuration set similar to *M* and the upper approximation represents the maximum optional configuration set similar to *M*.

#### 3.3.3. The Definition of the Division Matrix and Division Function in the Configuration Space

The division matrix of selected attributes *M* in the configuration decision tables is defined as follows:

(5) (Cij)={α∈M ∣ α(xi)≠ ∣ α(xj)} for  i,j=1,2,…,n.

The division function is defined as follows:

(6) $$\begin{array}{c} f\left(M\right)=\prod \sum_{\left(x,y\right)\in U\times U}\alpha \left(x,y\right). \end{array}$$

The division matrix and division function are used to infer the smallest reduction, which is a small subset of the attributes that can reflect implicit relationships in the selected configuration decision tables.

With the introduction of new technology or new systems, the relevant information is incompletely or vaguely stored, which leads to incomplete configuration space information. At this time, any attribute value field, *V*_*α*_, may contain unknown or missing attribute values, represented with an “∗.”

#### 3.3.4. The Similarity of the Configuration Alternatives

In the configuration alternatives decision table, SIM(*M*) is defined as

(7) SIM(M)={(x,y∈U∪U ∣ ∀a∈M,fa(x) =fa(y)  or  fa(x)=  ∗  or  fa(y)=∗)},

where SIM(*M*) is a compatible relationship; there is no distinction between any two configuration collections through a variety of attribute values.

*S*_*M*_(*x*) represents a set of configuration alternatives, similar to a configuration:

(8) $$\begin{array}{c} S_{M}\left(x\right)=\left\{y\in U\;|\;\left(x,y\right)\in \text{SIM}\left(M\right)\right\}. \end{array}$$

Generalized decision function ∂_*A*_(*x*) is as follows:

(9) $$\begin{array}{c} \partial_{A}\left(x\right)=\left\{f_{d}\left(y\right)\;|\;y\in S_{M}\left(x\right)\right\}. \end{array}$$

In the incomplete configuration decision table, the role of ∂_*A*_(*x*) is to relax the evaluation rating requirements of the performance of the configuration alternatives, which might include multiple decision attributes.

#### 3.3.5. Calculation of Determined Rules of the System Configuration Optimization

Any configuration rules where *t* → *d* (where *t* is a conditional attribute value and *d* is the decision attribute value) are called the determination rules, only if *t* → *d* is unambiguous in *S* and ||*t*||⊆||*d*||.

For any configuration in *S*, *t* → *d*  is determined, leaving no other condition attribute subset to determine the decision attribute value *d* in values *t*, which is to say the configuration rule *t* → *d* is determined.

For any configuration alternatives *x* ∈ *U* and *I*_*A*_(*x*)⊆*I*_{*d*}_(*x*), Δ_*U*_(*x*) is a division function only if Δ_*U*_(*x*) = ∏_*y*∈*Y*_∑*α*(*x*, *y*), where *Y*_*U*_ = *U*/*I*_{*d*}_(*x*) and Δ_*U*_(*x*) is a function of the determination of the configuration rules. We can get the optimization determination of configuration rules of the decision table through the establishment and reduction of the function.

#### 3.3.6. Calculation of Generalized Rules of the System Configuration Optimization

Δ_*g*_(*x*) is a determined division function of *x* (*x* ∈ *U*), only when Δ_*g*_(*x*) = ∏_*y*∈*Y*_∑*α*(*x*, *y*).

Where *Y*_*g*_ = *U*∖{*y* ∈ *U* | *d*(*y*) ∈ ∂_*AT*_(*x*)} and Δ_*g*_(*x*) is a function of the optimization of generalized configuration rules. We can get the optimization of generalized configuration rules from the decision table through the establishment and reduction of the function.

#### 3.3.7. Fuzzy C-Means Algorithm

We use the* fuzzy C-means* method to discrete the continuous data. The definition of FCM is summarized as follows:

 
*X* = {*x*_1_, *x*_2_,…, *x*_*n*_}, sampling set of an attribute,
 
*x*_*j*_ = (*x*_*j*1_, *x*_*j*2_,…, *x*_*jk*_), *j*th *k*-dimensional vector of each attribute,
 
*c*, the number of clusters that are specified,
 
*v*_*i*_, the center of the *i*th cluster,
  (10) $$\begin{array}{c} v_{i}=\frac{\sum_{j=1}^{n}{\left(u_{ij}\right)}^{q}x_{j}}{\sum_{j=1}^{n}{\left(u_{ij}\right)}^{q}}, \end{array}$$
 
*V* = (*v*_1_, *v*_2_,…, *v*_*c*_), center vector composed of a cluster center,
 
*q*, real number greater than 1,
 
*u*_*ij*_, weight index which control the fuzziness of the attribute clustering,
 
*ε*, termination condition determined by the engineering staff,
 ||*x*_*j*_−*v*_*i*_||^2^, Euler distance of *j*th attribute and the cluster center.

The definition of the membership function of each attribute vector to each attribute cluster is

(11) $$\begin{array}{c} u_{ij}=\frac{{\left[1/{||x_{j}-x_{i}||}^{2}\right]}^{1/\left(q-1\right)}}{\sum_{k=1}^{c}{\left[1/{||x_{j}-x_{k}||}^{2}\right]}^{1/\left(q-1\right)}}. \end{array}$$

In the process of discretization of continuous data, the minimal value of the following objective function is required:

(12) $$\begin{array}{c} J_{q}\left(u_{ij},v_{k}\right)=\sum_{j=1\;}^{n}\sum_{i=1}^{c}{\left(u_{ij}\right)}^{q}{||x_{j}-v_{i}||}^{2};\;c\leq n. \end{array}$$

The application procedures are summarized as follows.

**Step 1**

Determine the target that needs to be analyzed and the related attributes that need to be discretized.

**Step 2**

Determine a set of sampling points of the configuration attributes *X* = {*x*_1_, *x*_2_,…, *x*_*n*_} and *j*th *k*-dimensional vector of each attribute's sampling point.

**Step 3**

After discretization of the configuration attributes, allocate the value of *c*, *q*,  and *ε*.

**Step 4**

Initialize the membership function matrix *u*_*ij*_^0^, which represents the distance of each configuration attribute point to the initial cluster center.

**Step 5**

Use *u*_*ij*_^0^ and *v*_*i*_ to upgrade the center of each configuration property cluster.

**Step 6**

Calculate *u*_*ij*_^(*L*+1)^, which represents the relationship of each configuration attribute point to its center.

**Step 7**

If max⁡[||*u*_*ij*_^(*L*)^ − *u*_*ij*_^(*L*+1)^||] ≤ *ε*, then stop iteration; otherwise return to Step 5.

### 3.4. SOM-Based Exploration Algorithm

After the C-space area of concern is determined, using the SOM method, the configuration space is mapped to part of the design space, and the subsequent optimization is then capable of meeting the design specifications and requirements only in the area of concern.

SOM is an unsupervised learning neural network, which is a type of data clustering and high-dimensional data visualization method. The purpose of visualization is to project data onto a graphical representation to provide a qualitative idea of its properties. Typically, the multidimensional data is mapped to the two-dimensional space with hexagonal grids. Therefore, SOM further maps the configuration space region to the smaller design space area, which is the area of concern in the design space. Unlike conventional geographical methods, SOM cannot provide any geographical features, coordinates, distances, and so on, but it can describe closeness or distribution of the input design variables. After the initial aerospace system configuration is determined, the input layer of the *n*-dimensional design variables and the *m*-objective function as an input vector can be determined, where *n* and *m* are positive integers. The *n* + *m* neurons can then be assigned. In the output layer, the *n* + *m* dimensional weight vector = {*v*_1_, *v*_2_, *v*_3_,…, *v*_*n*+*m*_} is randomly assigned to neurons.

In SOM, unsupervised learning clusters similar patterns together, while preserving the topology of the input space and maintaining a full connection of the input vectors to neurons in the output layer. There are two main goals to be achieved. The first is that the output layer searches for the winning unit with a closer weight vector to each input vector.

The second is that, in order to be closer to the input design variables and objective function vectors, weight vectors of the winning unit and its neighboring neurons will be updated. As a result, the *n* + *m*-dimensional input vectors are projected onto a sequence of neighboring neurons in the two-dimensional hexagonal grid. From the color of the neurons in the output layer, we can compare the change trends of design variables or the correlation between design variables and objective functions.

The detailed steps of SOM application are summarized as follows.

**Step 1**

Assign the weight vector *V* = {*v*_1_, *v*_2_, *v*_3_,…, *v*_*n*+*m*_}.

**Step 2**

Select *n* design variables and *m*-objective functions as the input vectors.

**Step 3**

Get the neuron that has the least distance from input vectors.

**Step 4**

Update the weight vectors of the winning unit and its neighboring neurons.

**Step 5**

If the predefined iterative requirement is satisfied, stop. All the design variables and objective functions are projected onto the two-dimensional hexagonal grid. Otherwise, go to Step 2.

## 4. Case Study

### 4.1. Problem Description

In order to better demonstrate this method, a simple example problem will be used. This illustration is adapted from an example previously published by Griendling [16]. Note that the example is not designed to reflect reality, in order to avoid publication restrictions. The SEAD mission demonstrated the need for CBAs to explore a broad range of operational and materiel solutions. The considered alternatives included variations on operations, systems, organizational responsibilities, network structure, interoperability level, and force structure. Since the total alternative space had over 700,000,000 feasible architectures, it was decided to first group the alternatives by their system portfolios and eliminate portfolios with overall poor performance.

### 4.2. Parameter Settings

The following several alternatives were selected from numerous architecture alternatives as the basis for the aerospace SoS configuration. After processing the corresponding attribute values, the list was compiled, as shown in Table 1.

### 4.3. Experimental Results

Using the standard rough set theory for data mining, the continuous data should be discretized. In order to facilitate attribute processing, the attribute set *C* is divided into three categories. Among them, the first category includes *C*_1_ (cost) and *C*_2_ (time), the second category *C*_3_ (risk) and *C*_4_ (support level), and the third category *C*_5_ (P-success). The first class of continuous attributes is discrete with equal interval division, the attribute values of the second class use the range standardized management approach to discrete data and the third class attribute values are directly converted to discrete data.

Therefore, the attribute *C*_1_ is divided by 20 for each interval, *C*_2_ is discretized by 25 for each interval, and in *C*_3_, 1 represents general and 2 represents high. For attribute *C*_5_, 1 represents a success rate of 0.5 or more and 2 represents a success rate below 0.5. For attribute *C*_4_, 1 represents class I and 2 represents class II.

A sample attribute classification is shown in Table 2.

Calculated by the software* Rosetta*, the reduction of *C* by *D* can be obtained with {*C*_1_, *C*_2_}; the key of *C* is {*C*_1_, *C*_2_}.

The decision rules deduced from Table 2 are as follows.

**Rule 1**

*If C*_1_ = [80, 100)* and C*_2_ = [95, 120),* then* evaluation results = 1.

**Rule 2**

*If C*_1_ = [100, 120)* and C*_2_ = [95, 120),* then* evaluation results = 1.

**Rule 3**

*If C*_1_ = [80, 100)* and C*_2_ = [145, 170),* then* evaluation results = 1.

**Rule 4**

*If C*_1_ = [100, 120)* and C*_2_ = [95, 120),* then* evaluation results = 2.

**Rule 5**

*If C*_1_ = [120, 140)* and C*_2_ = [120, 145),* then* evaluation results = 2.

**Rule 6**

*If C*_1_ = [80, 100)* and C*_2_ = [170, 195),* then* evaluation results = 3.

**Rule 7**

*If C*_1_ = [140, 160)* and C*_2_ = [170, 195),* then* evaluation results = 2.

**Rule 8**

*If C*_1_ = [60, 80)* and C*_2_ = [45, 70),* then* evaluation results = 4.

**Rule 9**

*If C*_1_ = [60, 80)* and C*_2_ = [70, 95),* then* evaluation  results = 5.

**Rule 10**

*If C*_1_ = [80, 100)* and C*_2_= [70, 95),* then* evaluation results = 5.

**Rule 11**

*If C*_1_ = [80, 100)* and C*_2_ = [45, 70),* then* evaluation results = 6.

**Rule 12**

*If C*_1_ = [60, 80)* and C*_2_ = [70, 95),* then* evaluation results = 5.

Among which

 
Rule 1 and Rule 2 can be merged together:
   
*if C*_1_ = [80, 120)* and C*_2_ = [95, 120),* then* evaluation results = 1;
 
Rule 9, Rule 10, and Rule 12  can be merged together:
   
*if C*_1_ = [60, 100)* and C*_2_ = [70, 95),* then* evaluation results = 5.

Uncertainty rules are as follows.

**Rule 13**

*If C*_1_ = [100, 120)* and C*_2_ = [70, 95),* then* evaluation results = 3, and rule certainty factor is 0.5.

**Rule 14**

*If C*_*1*_ *=* [100, 120)* and C*_2_ = [70, 95),* then* evaluation results = 4, and the rule certainty factor is 0.5.

In the first mapping layer, the rules list which attributes have the greatest impact on the performance of the aerospace SoS.

Configuration rules show that cost and time are the core attributes of the decision table that influence the evaluation results.

In the process of aerospace SoS design or selection, the designer can select the satisfactory alternatives based on the extracted configuration rules, narrowing the range of options for candidate configuration alternatives.

In practical applications, decisions can be made according to the above rules of certainty and uncertainty.

After the first mapping, suppose that the designer needs to get the alternatives with evaluation results of 6. He can then choose configuration alternatives according to the rules *C*_1_ = [80,100) and *C*_2_ = [45, 70) and *C*_5_ = [0.6, 0.7), meaning that the costs should be between 80 and 100, time should be no more than 70 but not less than 45, and the task success rate will be between 0.6 and 0.7.

Before analysis with the SOM, a surrogate model must be established to approximately express the relationship between the variables and objective functions. Sampling 100 sets of data from the existing simulation database using the* Latin hypercube* experimental method, a neural network surrogate model must be established, using SOM to analyze the relationships between the objective function and design variables.

**Figure 3:**
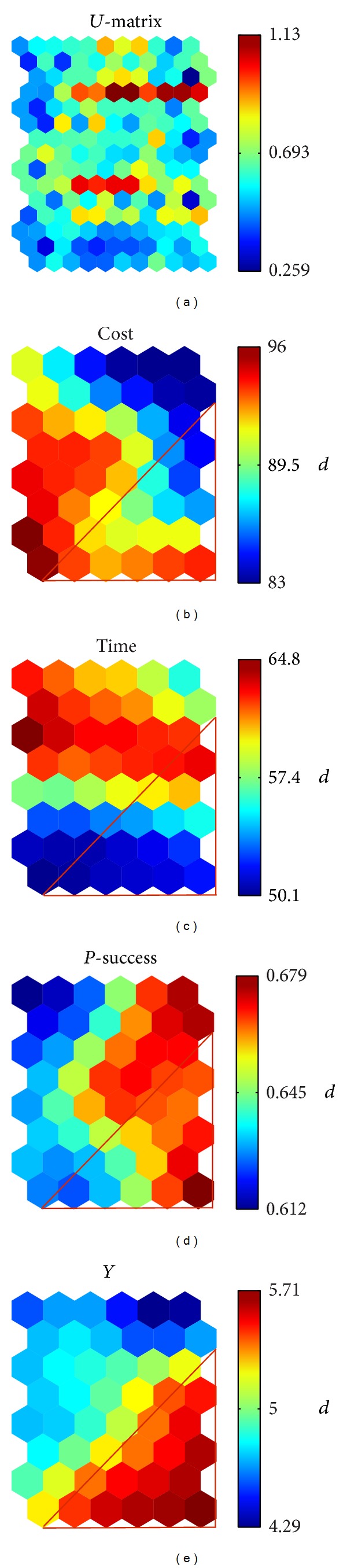
The SOM result I.

**Figure 4:**
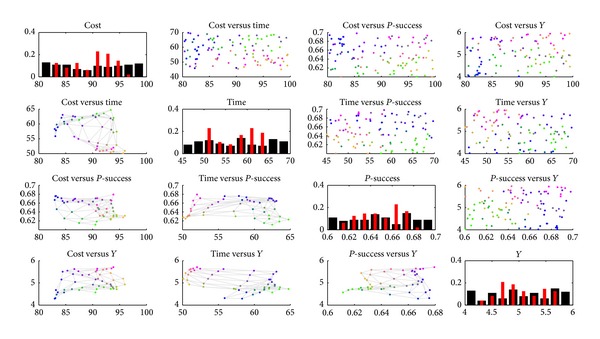
The SOM result II.

Figures 3 and 4 show the results of the analysis, using the SOM method. *Y* represents an optimized objective function (the highest evaluation value).

Objective function *Y* focuses on the right bottom of the graph; the costs graph is concentrated in the left corner.

For the sake of a bigger value of *Y*, as the red triangle in Figure 3(e), more attention should be paid to the corresponding red triangle in Figures 3(b), 3(c), and 3(d).

In this way, the value range of P-success should be (0.645, 0.679), rather than (0.612, 0.679), the cost of area is reduced to (81, 93), and the value range of time is reduced to (45, 60).

In Figure 4, simple scatter plots and histograms of all variables are shown. Original data points are in the upper triangle, map prototype values are in the lower triangle, and histograms are on the diagonal: black for the data set and red for the map prototype values. The variable values have been denormalized.

Therefore, compared with the initial design space, the interval of design variables has largely narrowed.

## 5. Conclusions

In this paper, we studied capability-focused aerospace system of systems architecture alternative design space exploration problems with bilayer mapping. Our results suggest that the RST method can effectively map aerospace system performance space to the configuration space, while a different configuration space is mapped to different regions, efficiently narrowing the design range and providing new ideas for the quick selection of alternatives. At the same time, the SOM method can effectively map the configuration space of aerospace system of systems to the design space and reduce the design dimension or range. This allows the focus to remain on the areas of concern. The optimized efficiency of aerospace system of systems design is fundamentally improved and, as mentioned above, the proposed method effectively explores the design space, reducing the design space range. Starting with the initial stage of the aerospace system of systems design, the method is optimized in the conceptual design phase, sufficiently solving the problem of computing complexity and search difficulty.

## Figures and Tables

**Table 1:** The similar cases and corresponding data.

Alternative	Cost	Time	Risk	Support level	*P*-success	Evaluation results
1	99	112	High	I	0.67	1
2	110	110	High	I	0.55	1
3	95	150	General	I	0.71	1
4	108	108	General	II	0.52	2
5	125	125	General	II	0.49	2
6	86	190	High	II	0.67	3
7	146	192	High	II	0.68	2
8	108	90	General	II	0.71	3
9	60	65	General	II	0.72	4
10	74	79	General	II	0.80	5
11	102	80	General	II	0.66	4
12	94	94	High	II	0.54	5
13	80	45	High	I	0.61	6
14	66	78	General	II	0.59	5

**Table 2:** The classification of sample attributes.

*S*	*C*_1_	*C*_2_	*C*_3_	*C*_4_	*C*_5_	*D*
*S*_1_	2	3	2	1	1	1
*S*_2_	3	3	2	1	2	1
*S*_3_	2	5	1	1	1	1
*S*_4_	3	3	1	2	2	2
*S*_5_	4	4	1	2	2	2
*S*_6_	2	6	2	2	1	3
*S*_7_	5	6	2	2	1	2
*S*_8_	3	2	1	2	1	3
*S*_9_	1	1	1	2	1	4
*S*_10_	1	2	1	2	1	5
*S*_11_	3	2	1	2	1	4
*S*_12_	2	2	2	2	2	5
*S*_13_	2	1	2	1	1	6
*S*_14_	1	2	1	2	2	5
